# Fault Identification Model Using Convolutional Neural Networks with Transformer Architecture

**DOI:** 10.3390/s25133897

**Published:** 2025-06-23

**Authors:** Yongxin Fan, Yiming Dang, Yangming Guo

**Affiliations:** 1 School of Computer Science, Northwestern Polytechnical University, Xi’an 710072, China; fanyongxin@mail.nwpu.edu.cn; 2 School of Electronic Engineering, Beijing University of Posts and Telecommunications, Beijing 100876, China; 3 School of Cybersecurity, Northwestern Polytechnical University, Xi’an 710072, China; yangming_g@nwpu.edu.cn

**Keywords:** fault identification, convolutional neural network, transformer

## Abstract

With the advancement of industrial manufacturing and the shift toward high automation, machines have increasingly taken over many production tasks, greatly improving efficiency and reducing human labor. However, this also introduces new challenges, particularly the inability of machines to autonomously detect and diagnose faults. Such undetected issues may cause unexpected breakdowns, interrupting critical operations, leading to economic losses and potential safety hazards. To address this, the present study proposes a novel hybrid deep learning framework that integrates Convolutional Neural Networks (CNN) for feature extraction with Transformer architecture for temporal modeling. The model is validated using NASA’s CMAPSS dataset, a widely used benchmark that includes multi-sensor data and Remaining Useful Life (RUL) labels from aeroengines. By learning from time-series sensor data, the framework achieves accurate RUL predictions and early fault detection. Experimental results show that the model attains over 97% accuracy under both single and multiple operating conditions, highlighting its robustness and adaptability. These findings suggest the framework’s potential in developing intelligent maintenance systems and contribute to the field of Prognostics and Health Management (PHM), enabling more reliable, efficient, and self-monitoring industrial systems.

## 1. Introduction

With the advancement of industrial intelligence, mechanical equipment has become increasingly prevalent in manufacturing, energy, transportation, and other fields. However, long-term operating equipment inevitably experiences performance degradation and unexpected failures, which not only affect production efficiency but may also lead to serious safety incidents. Therefore, developing efficient and accurate fault identification methods is significant for achieving predictive maintenance and reducing operational costs [1].

Traditional fault diagnosis methods can be primarily categorized into three types: physics-based methods, data-driven methods, and hybrid methods. Physics-based methods simulate fault characteristics by establishing dynamic or thermodynamic equations of equipment, offering clear physical interpretability. Reference [2] proposed a thermodynamic modeling approach requiring no additional sensors, using only externally measurable parameters to monitor performance degradation trends in gas turbine engines, introducing physically meaningful indices like heat loss index and power deficit index as performance evaluation metrics. However, accurate modeling of complex equipment often proves extremely challenging and computationally expensive. Data-driven methods employ machine learning to identify fault patterns from historical data, where convolutional neural networks (CNN) excel at local feature extraction while long short-term memory networks (LSTM) can model temporal dependencies. Reference [3] introduced a CNN model incorporating multi-scale convolution and adaptive weights, significantly improving fault diagnosis accuracy. Reference [4] combined residual networks with LSTM to achieve intelligent detection of engine performance anomalies. While data-driven methods do not require complete understanding of complex internal mechanisms, they do demand collection of substantial effective degradation data from physical systems. Hybrid methods attempt to combine advantages of both approaches but often face challenges like high model complexity and limited generalization capability in practical applications [5,6]. Moreover, they typically require extensive knowledge and experience about the physical systems, making data-driven methods with better generalization capability often preferred for fault identification tasks.

Recent advancements in deep learning provide new solutions to these challenges. While CNNs possess strong local feature extraction capabilities, their fixed receptive fields limit long-range dependency modeling. Transformers can effectively capture global relationships through self-attention mechanisms but may overlook fine-grained local features [7]. This complementary characteristic creates opportunities for developing more powerful fault identification models.

In addition to CNN- and LSTM-based methods, several graph neural network (GNN)-based fault diagnosis techniques have emerged in recent years. For instance, EG-SAGCN (Edge-guided Spatial-Attention GCN) learns adjustable edge weights when constructing the sensor graph and applies spatial attention to weight each neighbor’s features, thereby capturing both local and global relationships more precisely. POWGCN (Probability-Optimized Weighted GCN) incorporates prior probabilities to assign initial weights to graph edges and then dynamically updates these weights during training, improving robustness to noisy data and sparse labels. MMHGAT (Multi-Modal Hypergraph Attention Network) employs a hypergraph structure to treat multiple simultaneously changing sensors as a single hyperedge and uses attention mechanisms to fuse data from different modalities, effectively modeling high-order relationships among multiple channels and nodes. While these GNN-based approaches perform well in multi-sensor environments, they introduce additional computational overhead for edge-weight optimization or hypergraph construction and may rely heavily on accurate prior probability estimation.

By contrast, the CNN-Transformer architecture proposed in this paper first employs convolutional layers to reduce dimensionality and extract local features from sensor time-series data and then applies a Transformer’s multi-head self-attention module to capture global, long-range dependencies. This design not only efficiently learns fine-grained local patterns but also flexibly models long-distance correlations, achieving high classification accuracy while significantly reducing computational and memory overhead.

To address these needs, this paper proposes a general-purpose fault identification model based on CNN-Transformer hybrid architecture. The model extracts local spatiotemporal features from equipment sensor data through CNN while employing Transformer to model global dependencies across sensors and time, achieving effective fusion of multi-scale features. Tests conducted on aircraft engines (CMAPSS dataset) demonstrate outstanding performance in fault identification tasks, with excellent results under both single-condition and multi-condition scenarios.

The main contributions of this study include the following:

1. Propose a CNN-Transformer hybrid architecture that first uses convolutional modules to extract local temporal features and then employs the Transformer’s self-attention mechanism to capture global dependencies, achieving multi-scale information fusion.
2. Through joint hyperparameter experiments (window size, dropout, number of training epochs), we identify optimal configurations for different scenarios, ensuring both convergence speed and classification accuracy.
3. On the CMAPSS dataset, comparison with multiple baseline models demonstrates that the CNN-Transformer outperforms others under both single-condition and multi-condition settings, achieving over 98% in accuracy and F1-score.
4. We evaluate the model’s computational cost—including training duration and GPU memory usage—and show that the CNN-Transformer maintains high performance while significantly reducing resource consumption.

This research provides an effective solution for intelligent fault diagnosis of industrial equipment. Although current experiments primarily focus on aircraft engine datasets, the model architecture exhibits generalizability and can be extended to fault identification tasks for other industrial equipment.

## 2. Model Architecture

As a critical component in the aerospace field, fault diagnosis of aero engines is essential for ensuring flight safety and improving operational efficiency. Traditional fault detection methods primarily rely on physics-based modeling and rule-based analysis, which have inherent limitations. With the rapid development of data-driven approaches, new opportunities have emerged for intelligent and automated fault detection.

**Figure 1:**
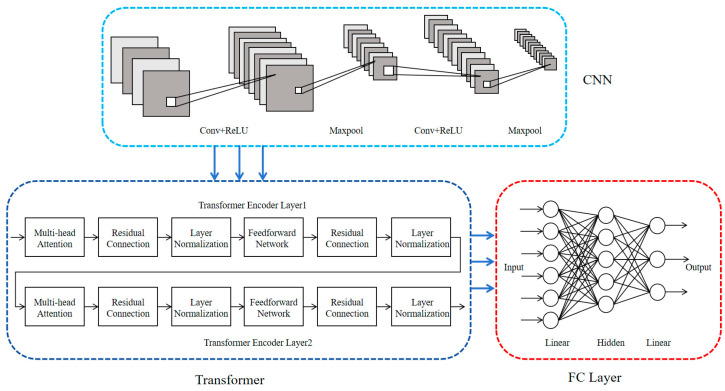
Architecture of the Fault Classification Model.

Contemporary deep learning approaches, particularly CNN-Transformer hybrids, demonstrate remarkable competence in handling multivariate temporal sequences. As illustrated in Figure 1, our architecture incorporates three core modules: convolutional blocks for spatial pattern abstraction, Transformer-based temporal dependency modeling, and regression-oriented dense layers. The computational workflow initiates with dual convolutional operations interleaved with pooling stages, establishing optimized feature hierarchy through spatial compression and local descriptor extraction. Before feeding the output into the Transformer encoder, a dimensional transformation is required. Specifically, a linear projection is applied to map the CNN-extracted features into a sequence of tokens suitable for Transformer input. After processing by the Transformer encoder, another linear layer is used to restore the original feature dimensions. Finally, a fully connected layer integrates the high-level semantic features and outputs the classification probabilities.

In this study, CNN is primarily used to extract local features from engine sensor data. By applying one-dimensional convolution operations, CNN maps the input time-series data into a high-dimensional feature space.

(1) $$Y=f_{c}\left(W\times X+b\right),$$

Here, Y denotes the convolution output, $X\in {\mathbb{R}}^{B\times T\times C}$ is the input signal (with a dimension of Batch size×step×channel), W represents the convolution kernel, b is the bias term, and $f_{c}$ is the nonlinear activation function (such as ReLU). The pooling operation further reduces the data dimensionality, enhances computational efficiency, and eliminates redundant information. In this study, max pooling is employed for data compression.

However, CNN primarily focuses on local patterns and has limited capability in capturing global information over long time series. To address this limitation, a Transformer encoder is introduced on top of the CNN to enhance the modeling of global temporal dependencies. The Transformer relies on a self-attention mechanism to compute dependencies between time steps.

(2) $$Attention\left(Q,K,V\right)=\mathrm{softmax}\left(\frac{QK^{T}}{\sqrt{d_{k}}}\right)V,$$

Here $Q\in {\mathbb{R}}^{L\times d_{k}},K\in {\mathbb{R}}^{L\times d_{k}},V\in {\mathbb{R}}^{L\times d_{v}}$ represent the query, key, and value matrices, respectively, where L is sequence length, and $d_{k}$ and $d_{v}$ are the dimensionalities of the key (query) and value vectors. The term $\sqrt{d_{k}}$ is used to scale the dot-product before the SoftMax to prevent large magnitude values, which stabilizes the gradient during training.

To further enhance the model’s capability in temporal modeling, this study adopts a multi-head attention mechanism, which enables the model to learn different temporal features in parallel from multiple subspaces.

(3) $$MultiHead(Y)=MHA=Concat(head_{1},\ldots ,head_{H})W_{O},$$

(4) $$head_{i}=Attention(QW_{i}^{Q},KW_{i}^{K},VW_{i}^{V}),$$

Here Concat() denotes the feature concatenation operation. In the multi-head attention mechanism, the input channels are split into smaller sub-channels and then restored to the original data dimension through concatenation. $W_{O}$ is the linear transformation matrix used to ensure consistency between the input and output dimensions. $W_{i}^{Q}$, $W_{i}^{K}$, and $W_{i}^{V}$ represent the linear projection matrices for the attention i ($i\in \left[1,H\right]$) head. H denotes the number of attention heads.

In addition, the Transformer also incorporates residual connections, a feed-forward network (FFN), and layer normalization to enhance feature representation and improve training stability. The residual connection is used to mitigate the vanishing gradient problem and helps the model retain the original information.

(5) Y′=Y+MHA,

Layer normalization is used to enhance the stability of the data. It is applied to each feature across the input, and transforms the input vector x into the normalized output y:

(6) $$y=\gamma \frac{x-\mu }{\sigma +\varepsilon }\beta ,$$

Here μ is the mean, σ is the standard deviation, and ε is a small constant to prevent division by zero. γ and β are learnable parameters used to rescale and shift the normalized features, enabling the model to retain strong expressive capabilities.

FFN applies nonlinear transformations to increase feature capacity, allowing the model to learn more complex patterns.

(7) $$FFN(X)=\max(0,XW_{1}+b_{1})W_{2}+b_{2},$$

Here $W_{1}\;\mathrm{and}\;W_{2}$ is the trainable weight matrix and $b_{1}\;\mathrm{and}\;b_{2}$ is the bias term. X denotes the input matrix to the FFN.

After the feature extraction, the fault classification module integrates the output of the Transformer through a fully connected layer and finally generates a probability distribution over classes using the SoftMax function.

(8) $$\hat{y_{i}}=P(y_{i}|x)=\frac{e^{z_{i}}}{\sum_{j=1}^{C}e^{z_{j}}},$$

Here $z_{i}$ denotes the dimension i of the network output, and $P(y_{i}|x)$ represents the probability that the input sample x belongs to the class i. i,j∈{1,2,…,C} and C is the total number of classes.

The model is trained using the cross-entropy loss function.

(9) $$L=-\sum_{i}y_{i}\log\left(\hat{y_{i}}\right),$$

Here $y_{i}$ is the one-hot encoded ground truth label, and $\hat{y_{i}}$ is the predicted probability. The Adam optimizer is adopted, and the learning rate follows a dynamic adjustment strategy to ensure training stability and avoid becoming stuck in local optima.

## 3. Data Processing

### 3.1. Dataset Overview

The dataset utilized in this research comes from the publicly accessible CMAPSS turbofan engine dataset, provided by NASA. This dataset models the entire degradation process of an engine, from normal functioning to failure, using a highly detailed simulation platform [8]. It is divided into four sub-datasets, as outlined in Table 1.

The dataset contains two types of engine fault modes. Among them, FD001 and FD002 include only a single fault mode—HPC degradation, while FD003 and FD004 involve a combination of HPC and Fan degradation. Regarding operating conditions, FD001 and FD003 operate under a single condition, meaning all engines in these subsets run in the same environmental setting. In contrast, FD002 and FD004 include six different operating conditions, indicating that engines in these subsets run under six distinct environments. Each dataset records the engine unit number, the number of operating cycles, three operational settings, and readings from 21 sensors.

**Figure 2:**
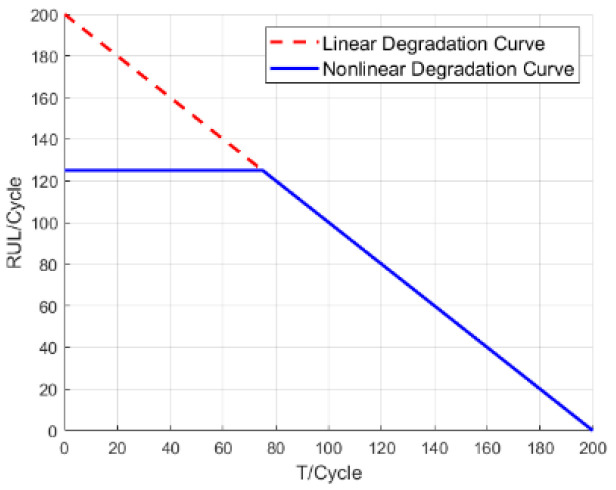
Piecewise linear degradation of RUL.

The CMAPSS dataset is widely used for Remaining Useful Life (RUL) prediction. In the early stage of engine operation, the RUL is relatively long, and the engine remains in a healthy state, during which sensor readings show minimal variation. Therefore, to obtain more effective mappings, many studies have adopted a piecewise linear degradation strategy [9,10,11], where the RUL is capped at 125. That is, when the actual RUL exceeds 125, the corresponding label is set to 125. The specific mapping relationship is illustrated in Figure 2.

In the early stage of engine operation, sensor readings show minimal variation, and the engine remains in a healthy state. This period, during which the RUL holds a constant value, is considered the fault-free phase. When the engine’s RUL falls below 125, sensor parameters begin to change, marking the onset of degradation. This linear degradation phase is regarded as faulty condition.

### 3.2. Data Preprocessing Under Single Operating Condition

Under a single operating condition, the influence of the environment on sensor parameters is minimal, and the changes are primarily driven by the engine’s internal state. Therefore, the focus of preprocessing lies in data denoising, normalization, and construction of time-series samples to ensure data quality and effective model learning.

In the CMAPSS dataset, some sensor readings exhibit very little variation throughout the entire engine cycle and thus provide limited useful information. Additionally, certain sensors may contain outliers due to measurement errors or hardware issues. To enhance data quality, we apply constant-sensor removal, outlier handling, and redundant feature filtering. Specifically, constant-sensor removal eliminates sensors whose values remain unchanged across the dataset, such as sensors 1, 5, 6, 10, 16, 18, and 19 [12].

Once the data have been denoised, we apply Z-score standardization to each retained sensor channel. Because each sensor originally operates on a different scale and unit, normalizing by subtracting the channel’s mean and dividing by its standard deviation (computed solely on the training set) places all features on a common zero-mean, unit-variance footing. This guarantees that no single sensor’s numerical magnitude will dominate the training process, thereby improving convergence speed and overall model performance.

**Figure 3:**
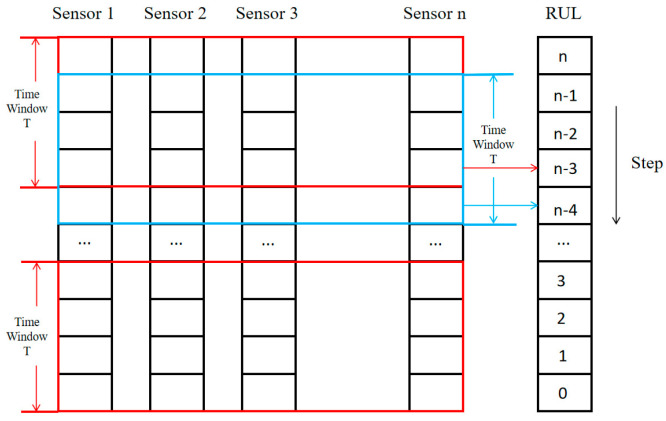
Illustration of the Sliding Window Mechanism.

The CMAPSS dataset records engine status on a cycle-by-cycle basis. However, sensor data from a single time point cannot effectively represent the operational trend of the engine. To address this, we adopt a sliding window approach to construct time-series input data, enabling the model to learn temporal features prior to fault occurrence, as illustrated in Figure 3. The window length is set to 90, and we will explain in Section 4.1 why a window size of 90 was chosen. The stride is set to 1, allowing overlapping between adjacent input samples and thereby enhancing the model’s ability to learn detailed features.

### 3.3. Data Preprocessing Under Multiple Operating Condition

In multi-condition scenarios, sensor parameters are influenced not only by the number of operating cycles but also by varying environmental conditions. This introduces additional complexity and disrupts the consistency of data patterns, significantly increasing the modeling difficulty.

#### 3.3.1. Clustering-Based Feature Processing

**Figure 4:**
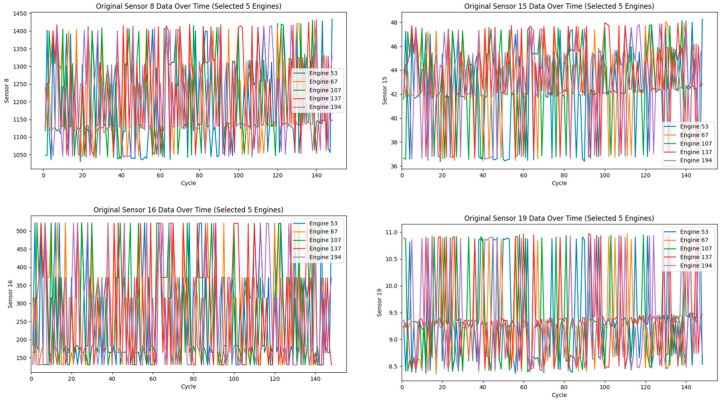
Sensor data without clustering and normalization.

Under multiple operating conditions, the sensor readings of a given engine may vary depending on the environment in which it operates. As a result, changes in sensor values are not solely driven by the number of cycles, leading to irregular and less predictable feature patterns. Figure 4 illustrates the variation in four sensor parameters from five engines with similar life spans. As shown, the feature trends of these sensors are not clearly distinguishable, making it difficult to establish accurate mappings.

**Figure 5:**
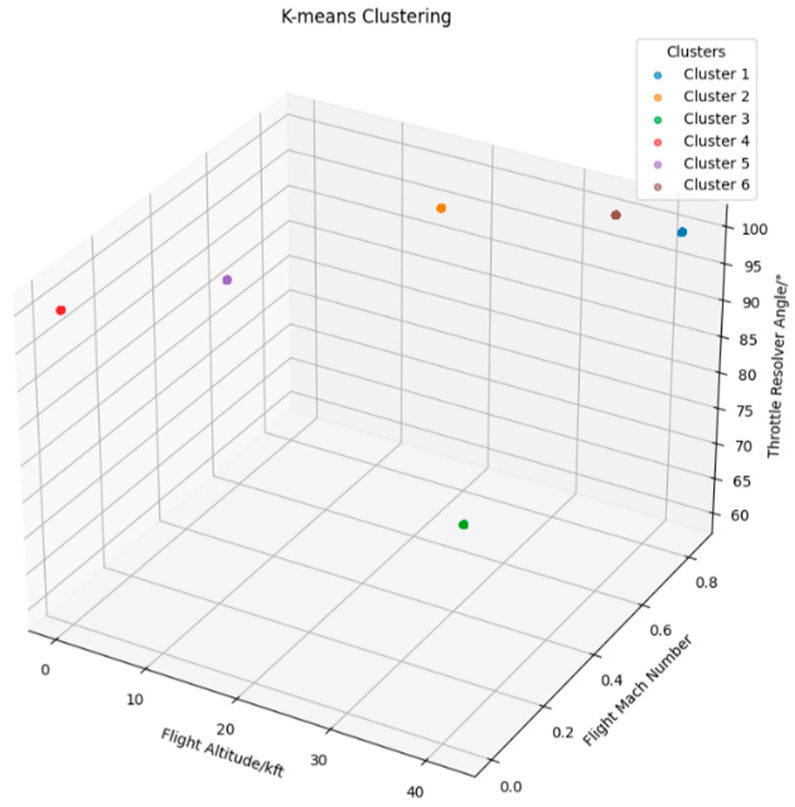
K-means clustering results in 3D space. The axes represent Flight Altitude (kft), Mach Number, and Throttle Lever Angle (°).

Therefore, effectively handling data under multiple operating conditions has become a key focus of research. To better reveal the degradation characteristics of sensor data over continuous flight cycles, we apply the K-means clustering algorithm to categorize the operating conditions [13]. The clustering results are shown in Figure 5. Table 2 lists the environmental parameters for the different operating conditions corresponding to the cluster numbers shown in Figure 5.

Based on the identified operating conditions, the mean and standard deviation of each sensor are calculated separately for each condition. Then, Z-score normalization is applied to each sensor’s data under its corresponding condition.

(10) $$X_{mn}^{\prime }=\frac{X_{mn}-\mu_{mn}}{\sigma_{mn}},$$

Here $\mu_{mn}\;\mathrm{and}\;\sigma_{mn}$ represent the mean and standard deviation of the sensor n under the operating condition m, and $X_{mn}$ denotes the corresponding raw value before normalization.

**Figure 6:**
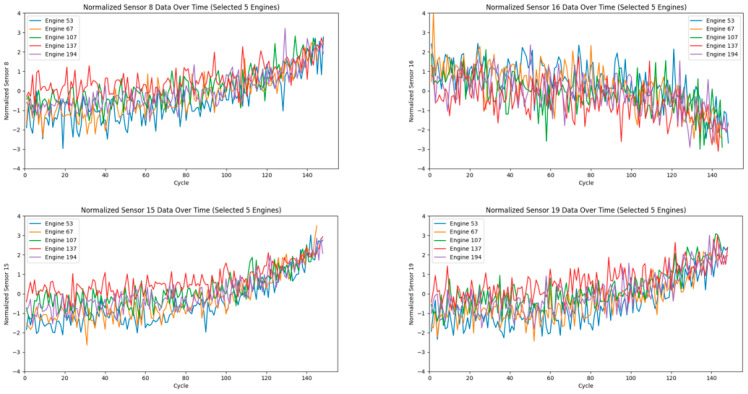
Sensor data after clustering and normalization.

After normalization, the variation trends of the sensor parameters become more distinct (see Figure 6), effectively enhancing the model’s ability to learn degradation patterns.

#### 3.3.2. Sensor Data Denoising and Sliding Window

After examining the sensor parameters post-normalization, some sensors still exhibited minimal variation. Upon further analysis, it was found that sensor 1 and sensor 18 showed almost no change. Therefore, these two sensors were removed, and the remaining 19 sensors were used as input features [14].

Additionally, to retain temporal dependencies in sensor data, a sliding window approach also was adopted, with a window size of 90 time-steps and a step size of 1. This ensures a continuous representation of system degradation trends and enhances model learning by capturing sequential fault patterns effectively.

### 3.4. Evaluation Metrics

To validate the effectiveness of the proposed model, five commonly used evaluation metrics are adopted: average precision, average recall, F1-score, accuracy, and loss:

(11) $$Precision=\frac{TP}{TP+FP},$$

(12) $$Reacall=\frac{TP}{TP+FN},$$

(13) $$F_{1}=2\times \frac{precision\times Reacall}{precision+Reacall},$$

(14) $$Accuracy=\frac{TP+TN}{TP+TN+FP+FN},$$

Within a binary classification framework, the confusion matrix components are operationally defined as follows: *TP* quantifies authentic positive predictions, *FP* captures type I errors where negative samples receive positive labeling, *TN* reflects valid negative determinations, while FN embodies type II errors involving misassigned positive specimens.

In a multi-class setting (e.g., with classes A, B, and C), evaluation metrics for class A can be computed by treating class A as the positive class and the other two classes (B and C) as the negative class, effectively converting it into a binary classification problem.

## 4. Experimental Results

The CMAPSS dataset is split into training sets, test sets, and test labels. The training set captures the entire operational history of an engine from start to complete failure, while the test set includes only a subset of flight cycles for each engine, simulating real-world data at unknown intervals. The associated test labels indicate the RUL of the engine. As a result, the test set cannot be directly applied to the fault classification task in this research.

For the single-condition scenario, the training sets from FD001 and FD003 are used as experimental datasets. For the multi-condition scenario, the training sets from FD002 and FD004 are adopted. We randomly split 70% of the data as the training set, 20% as the validation set, and the remaining 10% as the test set.

**Figure 7:**
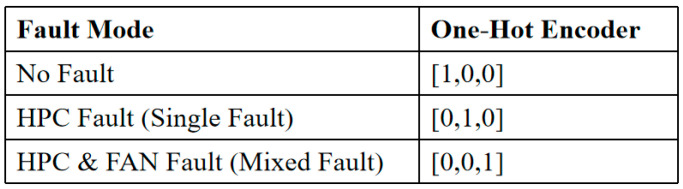
One-hot vector encoding.

In our model, categorical labels are transformed into one-hot encoded vectors to facilitate effective learning. Each fault type is represented as a unique binary vector, where only one element is set to 1 while the others remain 0. This approach ensures a clear and distinct representation of each class, enhancing the robustness of the classification process. Figure 7 shows the one-hot vector encoding used in this study.

### 4.1. Hyperparameter Experiment

During data preprocessing, since sensor parameters under multiple operating conditions may be affected by environmental variations, the input data dimensions differ from those under single-condition scenarios. As a result, the hyperparameter settings are also adjusted accordingly.

To determine the optimal hyperparameter combination for the CNN–Transformer model, we conducted joint experiments on Dropout, Epoch, and Window Size. During the experiments, we found that, with the learning rate and batch size held constant, the single-condition model usually fully converges by Epoch = 50, and sometimes even triggers early stopping in the low 40s. Therefore, for the single-condition scenario, we fixed Epoch at 50 and then examined the effects of Dropout and Window Size; similarly, in the multi-condition scenario, the model only fully converges when Epoch = 40, so we fixed Epoch at 40 before discussing the remaining two hyperparameters.

**Figure 8:**
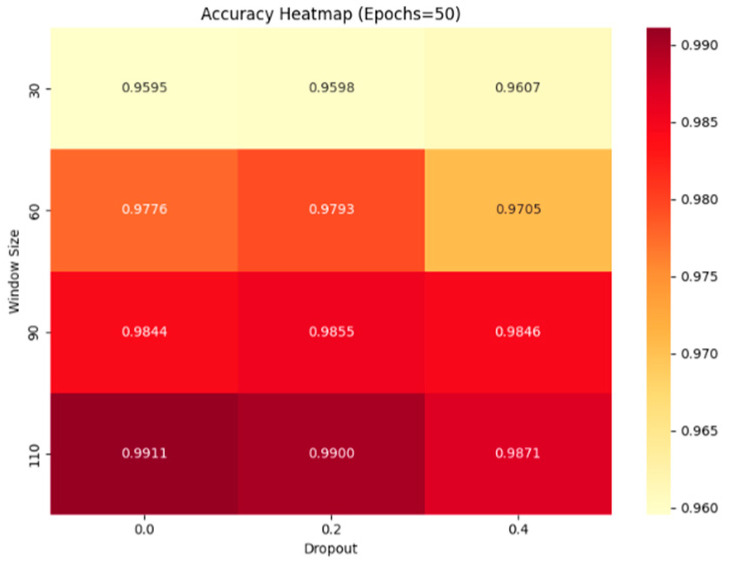
Accuracy heatmap under single scenario (Epochs = 50).

**Figure 9:**
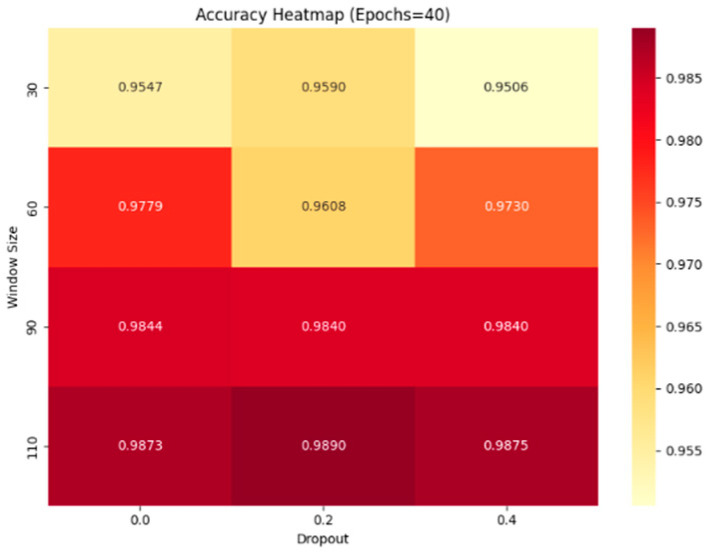
Accuracy heatmap under multiple scenario (Epochs = 40).

Figure 8 and Figure 9 show heatmaps of accuracy for different combinations of Dropout and Window Size under single-condition (Epoch = 50) and multi-condition (Epoch = 40) settings, respectively. As the figures reveal, accuracy peaks when the Window Size increases to 110. This is because the Transformer excels at modeling long-range dependencies: a larger window provides more contextual information for the self-attention mechanism, allowing the model to better extract features from critical time steps. However, since an engine’s lifetime is finite—across the four datasets, the shortest engine cycle is only 115 cycles—the Window Size cannot grow without bound. Moreover, excessively large windows drastically reduce the number of available samples, which can in turn weaken the model’s generalization ability. Balancing these considerations, we chose a Window Size of 90 to ensure both sufficient temporal information and an ample sample count.

Regarding the Dropout parameter, although the heatmaps show that accuracy is slightly higher at Dropout = 0.2, the difference is negligible. To prevent overfitting and meet the computational constraints of industrial deployment, we should retain some degree of regularization by setting Dropout to 0.2, thereby improving stability and generalization with minimal sacrifice in performance. The specific hyperparameters are listed in Table 3.

### 4.2. Model Performance Evaluation

The changes in loss, accuracy, and F1-score on the training and validation sets are shown in the figures. As seen from the graphs, the loss function consistently decreases and converges with increasing training epochs, while the accuracy steadily improves and approaches a high, stable value—indicating that the model is effectively learning and converging.

**Figure 10:**
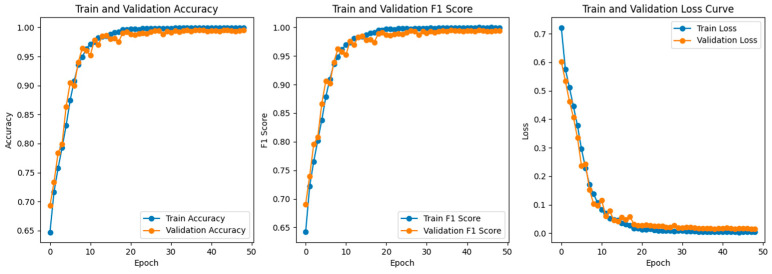
Training and Validation Accuracy, F1-Score, and Loss Curve under single operating condition.

**Figure 11:**
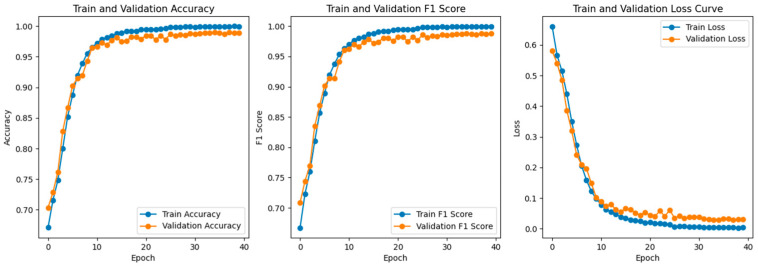
Training and Validation Accuracy, F1-Score, and Loss Curve under multiple operating conditions.

To further validate the model’s learning ability and stability under both single and multiple operating conditions, performance metric curves during the training and validation phases are plotted, as shown in Figure 10 and Figure 11. Figure 10 presents the accuracy, F1-score curves and loss curves of the training and validation under single-condition scenarios. Similarly, Figure 11 displays the corresponding metrics under multiple conditions.

Under single operating conditions, both accuracy and F1-score steadily increase with the number of training epochs and eventually stabilize. The final F1-score approaches 0.9896, indicating excellent classification performance. The loss function continues to decrease, and the training loss and validation loss closely align with minimal fluctuations, indicating good model stability.

Under multiple operating conditions, despite the increased complexity of data distribution, the model still exhibits strong learning ability. Accuracy and F1-score continue to improve during training and saturate around the 10th epoch, with F1-score consistently above 0.95. This indicates that the model maintains strong generalization capability even in complex scenarios.

Table 4 and Table 5 compare the performance of our proposed CNN Transformer with three baseline models—Vanilla Transformer, CNN-LSTM, and Time Net—under single and multiple operating conditions.

Under single operating conditions, the proposed CNN Transformer model significantly outperforms the three baseline models. Specifically, CNN Transformer reduces the loss to 0.0239, achieves 99.09% accuracy, and attains an F1-score of 98.96%. By comparison, the Vanilla Transformer—despite its self-attention mechanism—only reaches 96.62% accuracy and a 96.24% F1-score under the same data conditions. CNN-LSTM, due to its recurrent structure, captures temporal dependencies reasonably well but still lags the combined convolution-and-attention approach, achieving 93.76% accuracy and a 94.24% F1-score. Time Net, which relies solely on multi-scale convolutions without explicit global dependency modeling, obtains only 93.27% accuracy and a 94.02% F1-score. These comparisons indicate that for a relatively homogeneous, single-condition dataset—where environmental factors are stable—extracting local features via convolution followed by global self-attention allows the model to uncover deeper inter-sensor relationships, enabling rapid convergence to lower loss values and maintaining very high classification performance.

In the multi-condition scenario, the model must handle data distributions that vary across different operating environments. Although all models experience performance drops under this increased complexity, CNN Transformer still maintains a clear advantage, with loss equal to 0.0333, accuracy of 98.76%, and an F1-score of 98.54%. Vanilla Transformer’s accuracy and F1-score fall sharply to 88.14% and 87.81%, demonstrating that a purely attention-based mechanism struggles to capture local patterns accurately when distributions differ markedly. CNN-LSTM achieves 90.66% accuracy and a 90.83% F1-score in this scenario—better than Vanilla Transformer but still well below CNN Transformer—indicating that while recurrent units capture some temporal information, they do not represent long-range dependencies as effectively as multi-head self-attention. Time Net performs worst under multiple conditions, with only 72.19% accuracy and a 72.76% F1-score, proving that convolution-only architectures cannot handle cross-condition feature variation.

In summary, when the dataset includes multiple operating conditions, the CNN Transformer—by first using convolutions to extract local, time-ordered features and then applying Transformer self-attention to model global dependencies—adapts more robustly to drastic distribution shifts and continues to deliver superior classification performance in complex scenarios.

### 4.3. Model Overhead Experiment

Table 6 and Table 7 present a detailed comparison of computational overhead—measured by average training duration, peak GPU memory usage, and current GPU memory usage—for four competing models (CNN Transformer, Vanilla Transformer, CNN-LSTM, and Time Net) under both single and multiple operating conditions. Several notable patterns emerge from these results.

Under single operating conditions (Table 6), CNN Transformer requires an average of 1.47 s per training iteration, substantially less than Vanilla Transformer’s 1.88 s but somewhat higher than CNN-LSTM (0.93 s) and Time Net (0.77 s). This indicates that although the CNN Transformer integrates both convolutional and attention mechanisms, it remains more computationally efficient than a pure Transformer model in a homogeneous data setting. In terms of GPU memory consumption, CNN Transformer peaks at 52.61 MB and maintains a current usage of 23.61 MB—both figures are significantly lower than Vanilla Transformer’s peak of 125.3 MB and current usage of 32.34 MB. The convolutional front-end of the CNN Transformer effectively reduces the input dimensionality before feeding into the Transformer layers, thus curbing memory demands relative to the Vanilla Transformer. CNN-LSTM’s peak and current usages (83.01 MB and 23.48 MB, respectively) lie in between those of CNN Transformer and Vanilla Transformer, reflecting the intermediate overhead of recurrent units compared with pure attention. Time Net—being purely convolutional—exhibits moderate memory overhead (peak 65.78 MB, current 30.24 MB) but achieves the shortest per-iteration runtime (0.77 s) due to its simpler architecture.

When switching to multiple operating conditions (Table 7), all models incur greater overhead as dataset complexity increases. CNN Transformer’s average training duration escalates to 7.06 s, and its peak GPU memory usage rises to 83.68 MB (current usage 45.86 MB). Despite this increase, CNN Transformer still outperforms Vanilla Transformer—whose average iteration takes 7.40 s and peaks at 113.63 MB—highlighting that the CNN front-end continues to provide dimensionality reduction benefits even in heterogeneous scenarios. Compared to CNN Transformer, CNN-LSTM maintains the lowest runtime (4.12 s) and peak memory usage (70.59 MB), reflecting the relatively lightweight recurrence operations. However, CNN-LSTM’s reduced overhead comes at the cost of diminished classification performance under multi-condition settings. Time Net’s overhead (average 4.18 s, peak 84.95 MB, current 65.75 MB) is similar to CNN-LSTM’s, but its lower accuracy and F1-score in multi-condition experiments indicate that its simpler convolutional design cannot match the discriminative power of the more overhead-intensive CNN Transformer.

In summary, although multi-condition data inherently increases computational demands for all architectures, the CNN Transformer strikes the most favorable balance between performance and overhead. Its convolutional pre-processing mitigates memory consumption and accelerates training relative to a pure Transformer, while retaining superior classification accuracy compared to lightweight models like CNN-LSTM and Time Net.

## 5. Conclusions

In this work, we introduce a novel hybrid deep-learning framework for fault identification, which explicitly combines convolutional neural networks for local feature extraction with Transformer encoders for global dependency modeling. Unlike prior studies that rely solely on either CNNs (which lack long-range dependency capture) or pure Transformer architectures (which can overlook fine-grained temporal details), our approach fuses both architectures to exploit their complementary strengths. Concretely, the CNN front end reduces input dimensionality and highlights salient local patterns, while the Transformer’s multi-head self-attention layer learns global correlations among sensor readings over extended time spans.

To validate this design, we conducted comprehensive experiments on the standard CMAPSS turbofan-engine datasets under both single-condition (FD001 + FD003) and multi-condition (FD002 + FD004) scenarios. When compared against three strong baselines—Vanilla Transformer, CNN-LSTM, and Time Net—our CNN Transformer consistently outperformed all alternatives in classification accuracy and F1-score, while also exhibiting lower loss values. Under single-condition operation, CNN Transformer achieved 99.09 % accuracy and a 98.96 % F1-score. Even in the more challenging multi-condition setting—where engine data distributions vary significantly—our model retained 98.76 % accuracy and a 98.54 % F1-score. These quantitative gains demonstrate that integrating convolutional filtering with global self-attention not only improves fault identification performance but also enhances robustness to distributional shifts across operating regimes.

Beyond classification performance, we also evaluated computational overhead—measured by average per-iteration training time and GPU memory usage—for the four models. Under single-condition data, CNN Transformer achieved faster training (~1.47 s per batch) and lower peak GPU memory (52.6 MB) than Vanilla Transformer (1.88 s, 125.3 MB), due to its convolutional preprocessing reducing sequence length before attention. In multi-condition experiments; although CNN Transformer’s runtime increased (7.06 s per batch) to accommodate more complex data, it still required significantly less memory (83.7 MB peak) compared to Vanilla Transformer (113.6 MB peak) while delivering superior accuracy. These results confirm that our architecture strikes a favorable balance between interpretability, accuracy, and computational efficiency, making it suitable for industrial-scale deployment.

In summary, the key contributions of this research are threefold:

1. Novel Hybrid Architecture: We propose an end-to-end CNN Transformer model that fuses local convolutional feature learning with global self-attention, enabling more effective capture of both short-range and long-range sensor dependencies than existing CNN-only or Transformer-only frameworks.
2. Demonstrated Superiority over Baselines: Through rigorous comparisons on CMAPSS datasets, CNN Transformer outperforms other state-of-the-art approaches (Vanilla Transformer, CNN-LSTM, Time Net) in both single and multi-condition fault identification tasks, improving accuracy by at least 2–6 % under homogeneous operating data and by over 8 % under heterogeneous data.
3. Balanced Performance and Efficiency: By leveraging CNN layers to reduce input dimensionality before attention, our model achieves lower GPU memory usage and faster training times than pure Transformer models, while still maintaining top-tier fault-detection performance—even when data conditions vary dramatically.

Looking ahead, this hybrid framework offers a versatile foundation for several extensions. First, because our design naturally supports multi-sensor fusion, it could be adapted to other industrial prognostics tasks (e.g., predictive maintenance in manufacturing or wind-turbine monitoring) with minimal modification. Second, further enhancements—such as lightweight Transformer variants or dynamic routing in convolutional layers—could push the trade-off between accuracy and computational cost even further. Finally, incorporating online learning or federated learning schemes may enable real-time adaptation to new engine models or operational fleets without centralized data collection. Collectively, these future directions underscore the practical value of CNN Transformer for building next-generation, intelligent maintenance systems with both high accuracy and scalability.

## Figures and Tables

**Table 1:** Overview of the CMAPSS Dataset.

Dataset	FD001	FD002	FD003	FD004
Training Set (Number of Engines)	100	260	100	249
Test Set (Number of Engines)	100	259	100	248
Operating Conditions (Scenarios)	1	6	1	6
Fault Modes	1	1	2	2

**Table 2:** Operating conditions corresponding to each cluster.

Condition Number	Flight Altitude (kft)	Flight Mach Number	Throttle Resolver Angle (°)
Cluster 1	42	0.84	100
Cluster 2	20	0.7	100
Cluster 3	25	0.62	60
Cluster 4	0	0	100
Cluster 5	10	0.25	100
Cluster 6	35	0.84	100

**Table 3:** Hyperparameter settings for single and multiple operating conditions.

Hyperparameter	Single Scenario	Multiple Scenarios
Learning rate	$5\times 10^{-5}$	$2\times 10^{-5}$
Batch Size	64	32
Dropout	0.2	0.2
Epoch	50	40
Window Size	90	90

**Table 4:** Comparison of model performance under single operating conditions.

Model	Loss	Accuracy	Precision	Recall	F1
CNN Transformer	0.0239	99.09%	98.96%	98.97%	98.96%
Vanilla Transformer	0.0919	96.62%	96.19%	96.29%	96.24%
CNNLSTM	0.1525	93.76%	94.89%	93.96%	94.24%
Time Net	0.1521	93.27%	94.02%	94.08%	94.02%

**Table 5:** Comparison of model performance under multiple operating conditions.

Model	Loss	Accuracy	Precision	Recall	F1
CNN Transformer	0.0333	98.76%	98.41%	98.67%	98.54%
Vanilla Transformer	0.3489	88.14%	87.71%	88.08%	87.81%
CNNLSTM	0.2276	90.66%	90.66%	91.15%	90.83%
Time Net	0.5374	72.19%	79.1%	74.42%	72.76%

**Table 6:** Comparison of model overhead under single operating conditions.

Model	Average Training Duration (s)	Peak GPU Memory Usage (MB)	Current GPU Memory Usage (MB)
CNN Transformer	1.47	52.61	23.61
Vanilla Transformer	1.88	125.3	32.34
CNNLSTM	0.93	83.01	23.48
Time Net	0.77	65.78	30.24

**Table 7:** Comparison of model overhead under multiple operating conditions.

Model	Average Training Duration (s)	Peak GPU Memory Usage (MB)	Current GPU Memory Usage (MB)
CNN Transformer	7.06	83.68	45.86
Vanilla Transformer	7.4	113.63	66.91
CNNLSTM	4.12	70.59	42.42
Time Net	4.18	84.95	65.75

## Data Availability

https://data.nasa.gov/dataset/cmapss-jet-engine-simulated-data (accessed on 20 March 2025).
